# Development of Physical Activity Competence Test Battery and Evaluation Standards for Korean Children

**DOI:** 10.3390/children9010079

**Published:** 2022-01-05

**Authors:** Yeon-Oh Han, Byung-Sun Lee

**Affiliations:** Health Physical Activity Institute, Subin Art Inn Buingding, 25 Eonjuro 159 Gil, Gangnamgu, Seoul 06024, Korea; shotace@khu.ac.kr

**Keywords:** children, physical fitness for health, fundamental movement skills, physical activity habits, physical activity attitude, test battery, standard

## Abstract

The purpose of this study is to develop a comprehensive and systematic method and standard for evaluating children’s physical activity competency as a solution to the problem of increasing child obesity rates due to a decrease in physical activity among children. This study was used a cross-sectional study design. A literature review and Delphi survey were conducted to develop children’s physical activity competency evaluation. The evaluation criteria were presented based on the measurement data of metropolitan area kindergarten students (228 subjects) on the 2016 winter vacation. Items in the evaluation of children’s physical activity competency test battery include health physical strength, basic movement skills, physical activity habits, and physical activity attitudes. Physical fitness for health consisted of muscle strength and endurance (sit-up), flexibility (sit and reach, trunk lift) and body composition (BMI). Fundamental movement skills consisted of mobility (run, hop, jump), stability (static balance, dynamic balance), and control (throw kick). Physical activity habits consisted of the amount of daily steps (steps), exercise time, screen time, and sleep time. Physical activity attitudes consisted of preference, enjoyment, and confidence. The evaluation criteria for child physical activity competency test battery were presented in five stages, divided by age and gender. With the developed evaluation of children’s physical activity competency, the overall level of physical fitness for health, fundamental movement skills, physical activity habits, and physical activity attitudes of kindergarteners in the metropolitan area could be confirmed, and standards were presented.

## 1. Introduction

The world population was 7.32 billion in 2015, of which the child population accounted for 26.0%. By 2060, the proportion of the child population is expected to decrease by 5.5%, reaching 20.5% [1]. The population of Korea was 51 million in 2015, accounting for 13.9% of the population of children, however, it was reported that it would decrease by 3.7% to 10.2% by 2060 [2]. Contrary to the decline in the child population, the childhood obesity rate is increasing. According to the 2019 Student health Examination of the Ministry of Health and Welfare (2021), the obesity group in children was 13.7% (16.6% for male students and 10.6% for female students), and the overweight group was 11.1% (11.5% for male students and 10.7% for female students) [3]. Although the amount of physical activity is emphasized in the curriculum of children, the amount of physical activity is gradually decreasing due to the decrease in opportunities for physical activity [4]. In addition, an increase in the childhood obesity rate leads to a decrease in children’s athletic ability and physical activity level, and the motivation for challenging activities decrease, making them passive in participating in daily life and social play activities [5].

Physical fitness is one of the main factors in body development. In the curriculum of children, it presents an important learning goal to improve children’s basic physical fitness through physical exercise and health areas and emphasizes the importance of improving children’s physical fitness [6]. Physical fitness is divided into physical fitness for health and performance-related physical fitness. Physical fitness for health consisted of factors related to the maintenance of healthy life of muscular strength and endurance, cardiopulmonary endurance, flexibility, and body composition. Performance-related physical fitness consisted of agility, power, and balance of factors related to the performance of sports or athletic skills [7]. Childhood is a time when athletic ability to control the body develops, and as physical factors such as muscle strength, agility, coordination, and sense of balance develop, it is necessary to evaluate the level of physical fitness [8] Childhood is a time when various forms of exercise coordination are formed through physical activity in terms of exercise development. However, it is difficult to evaluate the development of exercise cooperation as an evaluation tool for the measurement of physical fitness [9]. In childhood, it is difficult to evaluate performance-related physical fitness and focus on movement development. Movement helps explore the environment and acquire information and changes to sports-related technic after childhood [10].

Fundamental movement skills are an important component of the exercise development stage [11], and it has a positive effect on physical activity play and sports activities in childhood and adolescence [12]. In addition, the higher the competence of fundamental movement skills, the higher the participation rate of physical activity, and obesity can be prevented due to regular physical activity [13]. Although, if exercise development is delayed due to restrictions on opportunities for various perceptual experiences in childhood, it negatively affects adolescent cognitive and emotional development [14,15,16]. As such, fundamental movement skills are an influential tool for evaluating and predicting the motor ability of growing children [17,18], and it is also possible to predict physical activity habits in adolescence [19].

Health problems have been raised due to the lack of physical activity of children. For this reason, various countries and institutions are presenting guidelines for physical activity for growing children, and studies related to the physical activity of children are being actively conducted. According to a report by Activity Health Kids Canada (2011), only 9% of boys and 4% of girls meet the recommended criteria for physical activity [20], and In the National Association for Sport and Physical Education (2005), only 54% of children met their physical activity [21]. A study by Tremblay et al. (2016) presented comprehensive guidelines for sleep time, sedentary life, and physical activity that affect the growth and development of children and adolescents [22]. Various methods have been proposed to measure the overall physical activity habits of sleep, sedentary, and physical activity. In particular, the pedometer is known as a useful tool for measuring the amount of physical activity in children [23]. However, through the pedometer, the overall movement of the day can be identified, but it is difficult to grasp the intensity and duration of exercise. Self-reported questionnaires are efficiently used in various fields as an alternative method to the pedometer [24]. Childhood is a period of various physical development and formation of physical activity attitudes [23]. Physical activity attitude is important in that interest in physical activity form the motivation to participate in activities such as play, games, and sports [25], and confidence in physical activity gained in childhood affects throughout life [26]. Therefore, it is significant to form a positive attitude toward physical activity in childhood. In order to voluntarily and actively participate in physical activities and form healthy physical activity habits, it is necessary to understand children’s physical activity attitudes [27].

Childhood is a developmental stage in which physical and verbal development occurs, and in the child curriculum [21,28], physical activity evaluation is mostly conducted as an observation evaluation [29,30], whereas it is difficult to use as an evaluation method in the childhood educational field. Various studies have suggested guidelines for physical activity for children’s health [20,21,22], however studies that present guidelines or standards for lifestyle habits considering growth and development are insufficient. Therefore, novel measurement and evaluation methods are being developed as Delphi methods due to difficulties in comprehensive comparison and evaluation of a single intervention using measurement tools of various methods. Extracting effective essential factors for school-based physical activity and health promotion through four stages of literature search, expert survey, expert interview, and expert meeting [31]. Development of guidelines for children’s physical activity and screen time [32]. Development of proactive methods that can increase physical activity and minimize sedentary behavior to promote healthy living habits [33]. A questionnaire design that measures children’s various levels of physical activity [34]. A comprehensive evaluation method of physical activity intervention was developed, and standardization of execution, measurement, and evaluation results was provided [35]. The priorities, appropriateness, and feasibility of effective intervention to prevent childhood obesity were determined, and methods and outcome measures were developed [36]. As such, the Delphi method is being used importantly to develop comprehensive evaluation methods and measures of various factors by extracting necessary factors through literature research and verifying the necessity of the field for experts.

Therefore, this study aims to develop a comprehensive and scientific evaluation method of childhood physical fitness for health, fundamental movement skills, physical activity habits, and physical activity attitudes using the Delphi method, and to prepare evaluation criteria by presenting mean and standard deviation by gender and age. Through this, a sustainable physical activity development system can be established, and it is intended to be used to evaluate children’s physical activity competence in physical education for growth and development.

## 2. Materials and Methods

There are two research contents to be conducted in this study. First, the development of a method for children’s physical activity competency test battery, and second, the criteria for evaluating the level of children’s physical activity competency are presented. The study was conducted with the approval of research from the Institutional Review Board of Ewha Womans University (IRB 121-8).

### 2.1. Participants

**Figure 1:**
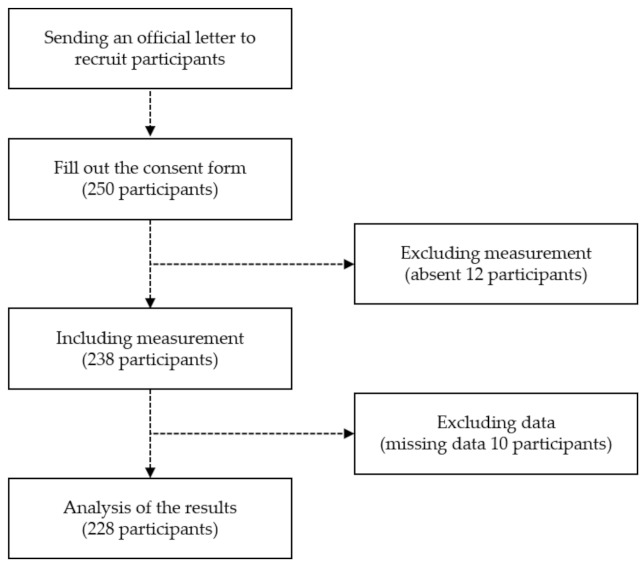
The process of recruiting participants.

An official letter was sent to children’s education institutions in major cities to recruit participants. The purpose of the study, experimental procedures, and potential risk factors that may occur during measurement were explained to institutional officials and parents who expressed their intention to participate. The experiment was conducted on subjects who prepared the consent form for participation in the experiment. In the case of diseases or disorders in the musculoskeletal system and vestibular organs, and if subjects, parents, and teachers did not want to, they were excluded from the experiment. Sufficient explanation and training were conducted to prevent musculoskeletal damage and injury when measuring physical fitness for health and fundamental movement skills. The process of recruiting subjects is shown in Figure 1.

The number of subjects to be measured in the physical activity competency evaluation was calculated under the condition of 10% tolerance. The subjects of reliability tests were 6 5-year-olds (3 males, 3 females), and 6 6-year-olds (3 males, 3 females), a total of 12. The subjects of evaluation standards development were 110 5-year-olds (59 males, 65 females), and 118 6-year-olds (51 males, 53 females), a total of 228. The characteristics of the subjects are presented in Table 1.

### 2.2. Development of Children Physical Activity Competence Test Battery

In order to develop the method of children’s physical activity competency test battery, domestic and foreign literature data were collected and analyzed on the factors of physical fitness for health, fundamental movement skills, physical activity habits, and physical activity attitude for children. Based on the collected domestic and foreign prior research data, researchers’ meetings, expert meetings, and expert surveys were conducted to select items that could be verified for validity and reliability and easily measured.

### 2.3. Preliminary Children Physical Activity Competency Test Battery and Method

Pre-measurement was performed to analyze the reliability of the measurement items. A total of 12 children aged 5 and 6 were randomly sampled from boys and girls, and the reliability test of the test-retest was conducted to measure the same as the draft test battery (10 days apart). The draft test was analyzed and confirmed as the final draft of children’s physical activity competency test battery in consideration of the reliability, validity, and ease of measurement through expert meetings.

### 2.4. Development of Children Physical Activity Competency Evaluation Standards

In the evaluation of child physical activity competency, descriptive statistical analysis was conducted on the measurements for each test item to present the mean and standard deviation by gender and age. The five-step criteria of physical activity competency evaluation were presented in five stages by applying percentages to each item of physical activity competency evaluation based on the criteria of the student health fitness evaluation system and Han et al. [37]. Stage 5 criteria were presented by dividing into phase 1 (very high, 5% or less), phase 2 (high, 6% or more to less than 35%), phase 3 (normal 35% or more to less than 65%), phase 4 (low, 65% or more to less than 95%), and phase 5 (very low, 95% or more).

### 2.5. Statistics

The results from this study were analyzed using SPSS PC^+^ for windows (version 20.0). The statistical methods conducted in this study are as follows. First, the CVR of 0.75 or higher for 8 experts and 0.56 for 12 experts satisfy the content validity at 95% confidence, and in a previous study [38]. In this study, content validity was investigated for 12 experts, and the significance level (a) of CVR was set to 0.56 or higher. Second, the test-retest of the developed draft physical activity competency test battery was measured, and Pearson’s correlation coefficient analysis was performed. The significance level (a) for reliability analysis was set to 0.05 and the correlation coefficient to 0.7 or higher. Third, the reliability test of the questionnaire survey (physical activity attitude) of the physical activity competency test battery was conducted. The Cronbach’s alpha coefficient was used as the reliability coefficient, and the significance level (a) for reliability analysis was set to 0.7 or higher. Fourth, the physical activity competency test battery developed for all participants was measured, and an independent *t*-test was conducted by dividing all test items by gender and age. The significance level (a) for independent *t*-test statistics analysis was set to 0.05.

## 3. Development of Children Physical Activity Competency Test Battery

In this study, the following five principles were applied to develop a method for evaluating children’s physical activity competency with reliability and validity.

First, physical fitness factors necessary in terms of growth and development in children are included.

Second, in terms of exercise development, large and small muscles are used, and fundamental movement skills necessary for children are included.

Third, it is composed of objective and not expensive to conduct a physical activity measurement, thereby increasing field utilization.

Fourth, it is configured to evaluate the attitude toward voluntary and active participation in physical activities in children.

Fifth, it consists of test items with a low risk of injury in evaluating physical fitness and fundamental movement skills.

According to the development principle of the evaluation method, domestic and foreign literature surveys were conducted, and the collected data were classified by each factor and expert Delphi surveys were conducted. The first Delphi survey was organized in the form of semi-structured questions, and the second survey was structured based on the results of the first survey. The selected measurement items were asked to respond to the appropriateness of the evaluation on a 7-point scale.

### 3.1. Development of Children Physical Activity Competency Test Battery Draft

In order to conduct the first Delphi survey, it was organized for each element and measurement item based on domestic and foreign literature on physical fitness for health, fundamental movement skills, physical activity habits, and physical activity attitudes. The first Delphi survey was conducted on 12 experts in early childhood education, measurement, and evaluation to select evaluation items. In addition, based on the results of the survey, an expert meeting was held to select items for evaluating children’s physical activity competency test battery. The factors of physical fitness for health are as follows. Sit-ups were recommended as a factor in muscle strength and endurance. As for the flexibility factors, bending in front of the upper body, shoulder flexibility, and lifting of the upper body were recommended. Body mass index was recommended as a factor in body composition. The factors of fundamental movement skills are as follows. Running, hopping, and jumping were recommended as mobility factors. Straight balance, rolling, and average walking were recommended as stability factors. Throwing, catching, hitting, and kicking were recommended as control factors. The factors of physical activity habit are as follows. Measurement of the number of steps using a pedometer was recommended for the factors of amount of physical activity. Measurement using a questionnaire tool was recommended for exercise time (time spent playing physical activities or sports activities above middle and vigorous intensity), screen time (time to sit or lie down because of the use of digital devices such as TV, computer, and mobile, excluding the purpose of learning), and sleep time. For the measurement of the factors of physical activity attitude, Brustad (1993) ‘Children’s Attraction to Physical Activity’ (CAPA) was reconstructed and used according to the contents of this study [25].

The second Delphi survey was conducted to select the most valid evaluation item among the inspection items selected through the first Delphi round. Twelve experts were asked to evaluate the appropriateness of the children’s physical activity competency test battery on a 7-point scale. The results of the second Delphi round are shown in Table 2.

Based on the evaluation results of the 2nd Delphi round, an expert meeting was conducted and the draft evaluation of children’s physical activity competency test battery was determined including physique (height and weight) measurement. The draft of the confirmed children physical activity competency test battery is presented in Table 3.

### 3.2. Reliability of Children Physical Activity Competency Test Battery

In order to evaluate the reliability of the draft child physical activity competency evaluation, the child physical activity competency evaluation was repeatedly measured twice 10 days apart. The participants of the preliminary measurement were 12 subjects similar to those of the evaluation standards development. As a result of analyzing the reliability of test-re-test of the measurement data, all tests except throwing and kicking showed high values of 0.7 or more. This means that repeated measurements of the same test can stably represent the same measurement value. In the case of throwing and kicking, the reliability coefficient was 0.7 or less. Throwing and kicking show relatively small deviations compared to other tests, and small deviations show low reliability for re-examination due to low variance between individuals. This is considered to be a characteristic of the throw and kick test and a characteristic of the subjects of the exercise development stage. It may be difficult to obtain stable measurements from all items for children, whereas all items showed a correlation coefficient above the normal level (0.7), and statistically significant results showed that all evaluation items for children were acceptable. The reliability analysis results of repeated measurements are shown in Table 4 and Table 5. The measurement methods of children’s physical activity competency test battery are presented in Table 6.

## 4. Result of Children Physical Activity Competency

Children’s physical activity competency test battery was measured with consent from 249 (boys: 110, girls: 118) children. The mean and standard deviation of physique, physical fitness for health, fundamental movement skills, physical activity habits, and physical activity attitude were presented.

The physique was measured for height and weight. There was a statistically significant difference between height (male = 0.000, female = 0.000) and weight (male = 0.000, female = 0.000) between ages within each gender, however only 5 years (height = 0.003, weight = 0.025) showed differences between genders within each age. Physical fitness for health was, measured by sit-ups, sit and reach, and trunk lift, and the body mass index was calculated using height and weight. Sit-up showed a significant difference between ages within each gender (male = 0.002, female = 0.000), trunk lift showed between ages within gender (female = 0.26). Physical fitness for health was measured by sit-ups, sit and reach, and trunk lift, and the body mass index was calculated using height and weight. Sit-up showed a significant difference between ages within each gender (male = 0.002, female = 0.000), trunk lift showed between ages within gender (female = 0.26). Fundamental movement skills measured mobility, stability, and control. Mobility was measured for running, hopping, and jumping, and stability was measured for static and dynamic balance. Manipulation measured throw and kick. Physical activity habits were measured through pedometers and questionnaires. The number of daily steps was measured using a pedometer, and exercise time, screen time, and sleep time were investigated using a questionnaire. The physical activity competency test battery measurement results are shown in Table 7.

## 5. Evaluation Standards of Children Physical Activity Competency

As for the evaluation criteria for child physical activity competency, the measurement results obtained in the study were used, and the five-stage relative evaluation criteria by gender and age were set. Stage 5 relative evaluation criteria were set as phase 1 (very high, 5% or less), phase 2 (high, 6% or more to less than 35%), phase 3 (normal 35% or more to less than 65%), phase 4 (low, 65% or more to less than 95%), and phase 5 (very low, 95% or more). In the case of the body mass index, it was set based on three-phase of underweight (low, 5% or less), normal weight (very high, 5% to less than 85%), overweight (85% to less than 95%), and obesity (95% or more) of the 2007 Korean Child and Adolescent Body Development Standard [39]. The criteria were set and presented to correspond to stage 1 for normal weight (very high), stage 3 (low) for underweight and overweight, and stage 4 (very low) for obesity. The evaluation criteria for child physical activity competency evaluation are presented in Appendix A (Table A1 and Table A2).

## 6. Conclusions and Suggestions

This study attempted to develop a more comprehensive, scientific, and utilization evaluation method for evaluating children’s physical activity, including physical fitness for health, fundamental movement skills, physical activity habits, and physical activity attitudes. In addition, by developing evaluation criteria for each gender and age based on the data obtained through measurement, it was conducted to establish a systematic childhood physical activity competency evaluation and improvement system, and the following conclusions were obtained.

### 6.1. Development of Children Physical Activity Competency Test Battery

The evaluation of children’s physical activity competency test battery developed through this study consisted of physical fitness for health, fundamental movement skills, physical activity habits, and physical activity attitudes necessary for childhood. For each measurement item, essential factors in childhood were selected through various domestic and international literature surveys. A plurality of test items corresponding to each factor were selected, and a 7-point scale evaluation was conducted on the Delphi survey and the appropriateness of the items. For test items that secured validity and reliability, test items that can be easily measured in the child education field were selected and developed as an evaluation of children’s physical activity competency test battery.

Through two test-retest and reliability analyses, the correlation coefficient between the first and second measurement results showed statistically significant (*p* < 0.05) results within the range of 0.687–0.991. As a result, it has a standard that can be used as a test battery to evaluate the physical activity competency of children in Korea and can be used as a method for evaluating the physical activity competency of children.

The finally selected test battery items for children’s physical activity competency evaluation consisted of preliminary investigation (physique), physical fitness for health, fundamental movement skills, physical activity habits, and physical activity attitudes. Height and weight are measured as a preliminary investigation of a child’s physique. Factors in terms of children’s physical development consisted of health and fundamental movement skills. There are four types of physical fitness for health: sit-up, sit and reach, trunk lift, and body mass index. Fundamental movement skills consisted of seven types: running, hopping, jumping, static balance, dynamic balance, throw, and kick. Factors in terms of children’s physical activity habits and emotional development consisted of physical activity habits and physical activity attitudes. The factors of physical activity habit consisted of four measurement items: daily steps, exercise time, screen time, and sleep time. As factors of physical activity attitude, three items of preference, enjoyment, and confidence were configured to be evaluated on a 5-point scale.

Physical fitness evaluation methods are widely used for elementary school students and above in Korea, however, no measurement and evaluation methods have been developed for children’s physical fitness and various physical activities. Factors evaluating physical fitness for health were composed of muscle strength and endurance, flexibility, and body mass index. Cardiopulmonary endurance items, an important factor in physical fitness for health, were excluded from this study. FITNESSGRAM, a tool for evaluating children’s health, does not recommend quantitative evaluation of children’s cardiopulmonary endurance using One-mile run and PACER, and the Walk test is classified as an event that is not applicable to children. It has been reported that children are not suitable for evaluation due to their difficulty in adjusting their pace and high dropout rate [40]. TGMD is a representative evaluation tool for child fundamental movement skills and consists of mobility and operability. It is reported that the standing on one foot of stability is an essential factor for all upright mobility technologies, and all mobility and operability require stability factors and are factors to be considered in the evaluation of motor skills [41]. In addition, stability is also included as an important factor in MABC-2 [42], PDMS [43].

The importance of physical activity habits and participation is emphasized, and various physical activity guidelines are recommended. In general, physical activity in daily life is measured by frequency, intensity, and period of physical activity [44]. Recently, the importance of sedentary life and sleep time, as well as physical activity, has been emphasized. The 24 h behavioral habits guidelines for children and adolescents include low-intensity physical activity, sleep, and sedentary life, and are also presented in the guidelines for physical activity for infants [22]. It was suggested that understanding children’s physical activity attitudes is necessary information to increase children’s participation in physical activities and to form active and healthy lifestyles [27]. In addition, the physical activity confidence formed in childhood is likely to last for a lifetime, and certain attitudes of this period are similar to previous studies’ suggestions that it can affect the exercise skills acquisition, physical health, and physical strength in the subsequent growth process [26]. Therefore, considering the contents of previous studies and the Delphi method conducted in this study, it is judged that evaluation factors consisting of children’s physical activity competency evaluation batteries’ health physical fitness, basic movement skills, physical activity habits, and physical activity attitudes are appropriate.

### 6.2. Development of Evaluation Standards for Children Physical Activity Competency

Measurements were conducted on boys and girls aged 5 and 6 using the child physical activity competency test battery developed through this study. Physical activity competency test battery was conducted on kindergarteners in major cities, and the number of subjects was calculated in consideration of the following conditions. First, the number of samples from similar previous studies [44,45]. Second, securing reliability, validity, and stability of evaluation tools (at least 5-fold the number of questions or at least 200 cases) [46,47]. Third, the condition of 95% confidence level and 10% allowable error level. Based on the 24 questions of the children’s physical activity competency test battery developed in this study, 240 children were selected and measured as final subjects in consideration of the dropout rate of 20%.

Descriptive statistical analysis of the measurement data was performed to obtain the mean and standard deviation according to item, age, and gender. There was a statistically significant difference according to age and gender. Accordingly, basic data on the system for comprehensive and scientific management of children’s physical activities were presented by presenting a physical activity competency test battery and evaluation standards by age and gender that can comprehensively evaluate children’s physical fitness for health, fundamental movement skills, physical activity habits, and physical activity attitudes.

### 6.3. Suggestion

This study attempted to establish a system that enables diagnosis and evaluation by developing a comprehensive and systematic method and standard for evaluating children’s physical activity competency as a solution to the problem of reducing the physical activity of children and adolescents. A literature review at domestic and international and an expert Delphi survey were conducted, and the overall level was identified by measuring physical activity competency for children, and the criteria were prepared. Previous studies reported a high prevalence of chronic diseases when students’ physical activity levels are low and suggested that strategies are needed to increase students’ physical activity [48]. In addition, physical activity in children is essential to improving physical strength and reducing the risk of metabolic and cardiovascular diseases, however current physical activity and education policies to reduce obesity in children are insufficient to address the lack of physical activity in schools. For a successful physical activity and education policy to reduce obesity in children, individual factors should be focused, but it was suggested that the level of activity of students and the promotion of physical activity of teachers should be included, but it was difficult to confirm the achievement of accurate results [49].

If this study is based on multilateral motivation to spread nationwide and be used voluntarily by all child-related educational institutions, the developed methods, and standards for evaluating child physical activity competency are expected to be used in various ways developing strategies to improve physical activity and lifestyle in child-related education and research. In the follow-up study, it will be necessary to analyze the factors of physical activity competency necessary for children’s growth development and health promotion through a study on the relationship between physical activity competency and health, nutrition, growth, and development. In addition, it is considered that research is needed to simplify the constituent items by integrating fundamental movement skills and physical fitness for health-related to exercise ability. Finally, standardized research should be conducted by expanding the scope of regions and targets, and it is suggested that research will be conducted to establish standards that take into account the developmental characteristics of adolescents after childhood so that children and adolescents can improve their physique and physical fitness based on physical activity.

### 6.4. Limitation

In this study, in order to derive the constituent factors of children’s physical activity competency, it was limited to domestic and foreign literature analysis and expert content validity survey, and the relationship between health and growth and physical activity competency could not be analyzed. In addition, a scale for physical activity was presented, though evaluation of the nutritional domain and scale were not presented. In the guide for a healthy life of children, nutrition and physical activity must be accompanied, and it is considered necessary to develop a nutrition-related scale for future research. A comprehensive measure of physical activity and nutrition to be developed in the future will be able to evaluate and analyze various individual factors of children’s physical activity and will be effectively used to improve childhood obesity and lifestyle by setting physical activity and nutritional goals.

## Figures and Tables

**Table 1:** Characteristics of participants.

Gender	Ages	*n*	Height (cm)	Weight (kg)
Male	5	59	114.5 ± 3.94	15.9 ± 1.48
	6	51	120.1 ± 3.68	16.1 ± 1.73
Female	5	65	112.1 ± 4.98	15.4 ± 1.46
	6	53	118.6 ± 4.79	16.0 ± 1.73

**Table 2:** Results of the 2nd Delphi round.

Category	Item	Contents	Mean	SD	Positive Rate (%)	Median	Mode	Interquartile Rage	CVR
Physical fitness for health	Strength and endurance	Sit-up	6.4	0.79	100	7.0	7	6.0–7.0	1.00
	Flexibility	Sit and reach	6.6	0.67	100	7.0	7	6.0–7.0	1.00
		Trunk lift	6.3	1.22	91.7	7.0	7	5.3–7.0	0.83
	Body composition	BMI ^1^	6.7	0.65	100	7.0	7	6.3–7.0	1.00
Fundamental movement skills	Mobility	Run	6.6	0.67	100	7.0	7	6.0–7.0	1.00
		Hop	6.6	0.67	100	7.0	7	6.0–7.0	1.00
		Jump	6.6	0.51	100	7.0	7	6.0–7.0	1.00
	Stability	Static balance	6.8	0.39	100	7.0	7	7.0–7.0	1.00
		Dynamic balance	6.7	0.65	100	7.0	7	6.3–7.0	1.00
	Control	Throw	6.4	0.79	100	7.0	7	6.0–7.0	1.00
		Kick	6.3	0.78	100	6.5	7	6.0–7.0	1.00
Physical activity habits	Steps		6.8	0.45	100	7.0	7	6.3–7.0	1.00
	Exercise time		6.3	0.97	91.7	6.5	7	6.0–7.0	0.83
	Screen time		6.3	1.15	91.7	7.0	7	6.0–7.0	0.83
	Sleep time		6.3	0.78	100	6.5	7	6.0–7.0	1.00
Physical activity attitude	Preference		6.7	0.89	91.7	7.0	7	7.0–7.0	0.83
	Enjoyment		6.7	0.49	100	7.0	7	6.0–7.0	1.00
	Confidence		6.9	0.29	100	7.0	7	7.0–7.0	1.00

^1^ body mass index.

**Table 3:** Items for children physical activity competency test battery.

Category	Item		Accept
Physical constitution	Height		Essential
	Weight		Essential
Physical fitness for health	Strength and endurance	Sit-up	Essential
	Flexibility	Sit and reach	Essential
		Trunk lift	Essential
	Body composition	BMI ^1^	Essential
Fundamental movement skills	Mobility	Run	Essential
		Hop	Essential
		Jump	Essential
	Stability	Static balance	Essential
		Dynamic balance	Essential
	Control	Throw	Essential
		Kick	Essential
Physical activity habits	Steps		Essential
	Exercise time		Essential
	Screen time		Essential
	Sleep time		Essential
Physical activity attitude	Preference		Essential
	Enjoyment		Essential
	Confidence		Essential

^1^ body mass index.

**Table 4:** Reliability analysis results of children physical activity competency test battery.

Contents	*n*	Test		Retest		r	*p*	Acceptance
		Mean	SD	Mean	SD			
Sit-up	12	7.3	7.00	7.1	6.73	0.991	0.001	Accept
Sit and reach	12	11.2	3.50	9.9	3.45	0.902	0.001	Accept
Trunk lift	12	20.4	5.86	18.8	5.31	0.883	0.001	Accept
BMI ^1^	12	16.1	1.24	16.1	1.24	0.998	0.001	Accept
Run	12	6.2	1.67	6.9	2.10	0.944	0.001	Accept
Hop	12	5.2	1.85	5.0	1.32	0.743	0.006	Accept
Jump	12	111.8	18.64	108.5	18.05	0.944	0.001	Accept
Static balance	12	15.9	12.16	15.7	12.54	0.967	0.001	Accept
Dynamic balance	12	14.0	3.80	13.0	3.32	0.907	0.001	Accept
Throw	12	5.2	1.34	4.5	1.57	0.694	0.012	Accept
Kick	12	6.1	1.51	5.7	1.44	0.687	0.014	Accept
steps	12	5646.2	1374.54	5153.8	1502.79	0.810	0.001	Accept
Exercise time	12	34.3	30.08	30.0	17.53	0.921	0.001	Accept
Screen time	12	31.8	18.26	30.9	17.82	0.987	0.001	Accept
Sleep time	12	560.3	19.27	556.9	20.83	0.837	0.001	Accept
Preference	12	15.9	2.61	14.9	2.23	0.841	0.001	Accept
Enjoyment	12	16.0	2.80	14.9	1.98	0.838	0.001	Accept
Confidence	12	16.0	2.59	15.1	2.27	0.709	0.010	Accept

^1^ body mass index.

**Table 5:** Cronbach’s *a* analysis results of physical activity attitude.

Contents	Scale Mean if Item Deleted	Scale Variance if Item Deleted	Corrected Item- Total Correlation	Cronbach’s Alpha if Item Deleted	Cronbach’s *Alpha*
Preference 1	10.61	7.076	0.805	0.894	0.917
Preference 2	10.67	7.272	0.823	0.888	
Preference 3	10.70	7.125	0.857	0.876	
Preference 4	10.86	7.519	0.755	0.910	
Enjoyment 1	11.27	5.631	0.728	0.876	0.892
Enjoyment 2	11.42	5.865	0.815	0.844	
Enjoyment 3	11.64	5.757	0.719	0.879	
Enjoyment 4	11.35	5.883	0.802	0.848	
Confidence 1	10.80	7.523	0.764	0.890	0.908
Confidence 2	11.10	7.042	0.787	0.883	
Confidence 3	10.95	7.199	0.823	0.870	
Confidence 4	10.95	7.272	0.795	0.880	

**Table 6:** Measurement method of children physical activity competency test battery.

Category	Item		Contents
Physical fitness for health	Strength and endurance	Sit-up	Fold your knees and lie on the floor and curl up your upper body so that your fingertips touch your knees.
	Flexibility	Sit & reach	Sit with your legs straight, bend your upper body, and stretch both hands forward as much as possible.
		Trunk lift	Attach both feet, look at the floor, lie down, and lift your upper body as much as possible.
	Body composition	BMI ^1^	Measure height and weight and calculate BMI (kg·m^−2^).
Fundamental movement skills	Mobility	Run	Turn around the cone in time for the “start” signal and return to the starting point as quickly as possible.
		Hop	Hop eight times in a row on one foot and move as far as possible.
		Jump	Jump as far as possible with both feet at the same time.
	Stability	Static balance	Close your eyes according to the “start” signal and maintain your posture as long as possible with one foot.
		Dynamic balance	Move to the end of the beam as quickly as possible according to the “start” signal. Move so that the ends of the front and rear feet meet without running.
	Control	Throw	Stand at the starting point and throw the ball five times on the target point.
		Kick	Run from the starting point and kick the ball into the target point at the preparation point.
Physical activity habits	Steps		Measure the steps for a week using a pedometer (5 days during the week, 2 days during the weekend).
	Exercise time		The physical activity, play, and sports activity time of moderate to vigorous intensity are measured and recorded by parents.
	Screen time		Using digital devices such as TVs, computers, and mobile devices, the time spent sitting or lying down is measured and recorded by parents.
	Sleep time		Parents measure and record the time a child sleeps in the evening.
Physical activity attitude	Preference		Children and parents fill out the contents of the questionnaire together.
	Enjoyment		
	Confidence		

^1^ body mass index.

**Table 7:** Children’s physical activity competency.

Category	Contents	Gender	Ages	Mean	SD	Classification	*p*-Value	
Physique	Height (cm)	Male	5 (59)	114.5	3.94	Male	0.001	***
			6 (51)	120.1	3.68	Female	0.001	***
		Female	5 (65)	112.1	4.98	5 years	0.003	**
			6 (53)	118.6	4.79	6 years	0.088	NS
	Weight (kg)	Male	5 (59)	15.9	1.48	Male	0.001	***
			6 (51)	16.1	1.70	Female	0.001	***
		Female	5 (65)	15.4	1.46	5 years	0.025	*
			6 (53)	16.0	1.73	6 years	0.425	NS
Physical fitness for health	Sit-up (number)	Male	5 (59)	6.5	10.00	Male	0.002	**
			6 (51)	13.6	13.39	Female	0.001	***
		Female	5 (65)	5.4	7.58	5 years	0.478	NS
			6 (53)	13.3	10.98	6 years	0.922	NS
	Sit and reach (cm)	Male	5 (59)	9.2	4.45	Male	0.543	NS
			6 (51)	9.7	4.97	Female	0.086	NS
		Female	5 (65)	9.6	4.03	5 years	0.642	NS
			6 (53)	10.9	4.63	6 years	0.211	NS
	Trunk lift (cm)	Male	5 (59)	19.6	5.24	Male	0.068	NS
			6 (51)	21.4	4.84	Female	0.026	*
		Female	5 (65)	20.1	4.74	5 years	0.543	NS
			6 (53)	22.2	4.98	6 years	0.414	NS
	BMI (kg·m^−2^)	Male	5 (59)	15.9	1.48	Male	0.905	NS
			6 (51)	16.1	1.70	Female	0.439	NS
		Female	5 (65)	15.5	1.46	5 years	0.391	NS
			6 (53)	16.0	1.73	6 years	0.899	NS
Fundamental movement skills	Run (sec)	Male	5 (59)	5.7	0.61	Male	0.001	***
			6 (51)	5.3	0.45	Female	0.001	***
		Female	5 (65)	6.4	0.93	5 years	0.001	***
			6 (53)	5.7	0.45	6 years	0.001	***
	Hop (m)	Male	5 (59)	4.4	1.34	Male	0.001	***
			6 (51)	5.9	1.26	Female	0.001	***
		Female	5 (65)	3.9	1.14	5 years	0.022	*
			6 (53)	5.6	1.20	6 years	0.196	NS
	Jump (cm)	Male	5 (59)	104.5	14.46	Male	0.001	***
			6 (51)	114.8	14.33	Female	0.001	***
		Female	5 (65)	90.4	13.22	5 years	0.001	***
			6 (53)	102.5	13.98	6 years	0.001	***
	Static Balance (sec)	Male	5 (59)	8.18	6.84	Male	0.034	*
			6 (51)	11.0	7.15	Female	0.019	*
		Female	5 (65)	7.8	6.91	5 years	0.205	NS
			6 (53)	14.1	12.95	6 years	0.132	NS
	Dynamic balance (sec)	Male	5 (59)	15.6	7.34	Male	0.042	*
			6 (51)	13.15	5.58	Female	0.134	NS
		Female	5 (65)	15.8	5.81	5 years	0.972	NS
			6 (53)	13.8	7.70	6 years	0.579	NS
	Throw (score)	Male	5 (59)	2.9	2.44	Male	0.001	***
			6 (51)	4.6	1.91	Female	0.001	***
		Female	5 (65)	2.7	2.14	5 years	0.621	NS
			6 (53)	4.5	2.21	6 years	0.884	NS
	Kick (score)	Male	5 (59)	5.5	2.23	Male	0.004	**
			6 (51)	6.5	1.98	Female	0.018	*
		Female	5 (65)	5.2	2.73	5 years	0.625	NS
			6 (53)	6.3	2.19	6 years	0.455	NS
Physical fitness habits	Steps (step)	Male	5 (59)	5850.8	2070.75	Male	0.328	NS
			6 (51)	6205.5	1733.25	Female	0.205	NS
		Female	5 (65)	5706.1	1474.29	5 years	0.646	NS
			6 (53)	6048.9	1493.03	6 years	0.616	NS
	Exercise time (min)	Male	5 (59)	37.1	32.39	Male	0.941	NS
			6 (51)	37.4	13.82	Female	0.669	NS
		Female	5 (65)	34.8	21.37	5 years	0.632	NS
			6 (53)	36.5	22.65	6 years	0.793	NS
	Screen time (min)	Male	5 (59)	43.1	22.77	Male	0.530	NS
			6 (51)	40.5	20.79	Female	0.654	NS
		Female	5 (65)	44.5	22.81	5 years	0.731	NS
			6 (53)	42.6	25.10	6 years	0.648	NS
	Sleep time (mini)	Male	5 (59)	574.3	40.21	Male	0.004	**
			6 (51)	548.2	54.22	Female	0.370	NS
		Female	5 (65)	568.6	31.59	5 years	0.367	NS
			6 (53)	562.6	41.65	6 years	0.123	NS
Physicalfitness attitude	Preference (score)	Male	5 (59)	14.32	3.78	Male	0.908	NS
			6 (51)	14.4	3.48	Female	0.158	NS
		Female	5 (65)	13.8	3.41	5 years	0.424	NS
			6 (53)	14.7	3.51	6 years	0.650	NS
	Enjoyment (score)	Male	5 (59)	15.1	2.81	Male	0.901	NS
			6 (51)	15.2	3.30	Female	0.094	NS
		Female	5 (65)	14.9	3.31	5 years	0.626	NS
			6 (53)	15.9	3.16	6 years	0.291	NS
	Confidence (score)	Male	5 (59)	14.4	3.46	Male	0.171	NS
			6 (51)	15.3	3.45	Female	0.013	*
		Female	5 (65)	13.6	3.24	5 years	0.196	NS
			6 (53)	15.2	3.87	6 years	0.953	NS

BMI: body mass index; *: *p* < 0.05, **: *p* < 0.01, ***: *p* < 0.001, NS: non-significant difference.

## Data Availability

The data presented in this study are available on request from the corresponding author.

## Appendix A

**Table A1:** Physical Activity Competence Evaluation Standards.

Category	Contents	Gender	Years	Phase 5 (Very Low)	Phase 4 (Low)	Phase 3 (Normal)	Phase 2 (High)	**Phase 1 (Very High)**
Physical fitness for health	Sit-up (number)	Male	5	0	1	2~6	7~20	21 or higher
			6	0	2~5	6~17	18~41	42 or higher
		Female	5	0	1	2~4	5~22	23 or higher
			6	0	2~7	8~15	16~32	33 or higher
	Sit and reach (cm)	Male	5	1.0 or less	1.1~7.7	7.8~10.1	10.2~17.8	17.9 or higher
			6	1.4 or less	1.5~8.6	8.7~11.5	11.6~18.5	18.6 or higher
		Female	5	2.5 or less	2.6~7.9	8.0~11.7	11.8~17.9	18.0 or higher
			6	2.5 or less	2.6~9.5	9.6~12.2	12.3~20.4	20.5 or higher
	Trunk lift (cm)	Male	5	10.0 or less	10.1~17.3	17.4~20.9	21.0~27.9	28.0 or higher
			6	14.6 or less	14.7~19.5	19.6~22.9	23.0~29.9	30.0 or higher
		Female	5	9.9 or less	10.0~18.9	19.0~21.9	22.0~27.5	27.6 or higher
			6	12.9 or less	13.0~20.8	20.9~24.9	25.0~29.4	29.5 or higher
Fundamental movement skills	Run (sec)	Male	5	6.67 or higher	5.86~6.66	5.55~5.85	4.50~5.54	4.49 or less
			6	6.14 or higher	5.39~6.13	5.18~5.38	4.56~5.17	4.55 or less
		Female	5	9.11 or higher	6.50~9.10	5.97~6.49	5.43~5.96	5.42 or less
			6	6.56 or higher	5.79~6.55	5.57~5.78	4.94~5.56	4.93 or less
	Hop (m)	Male	5	1.9 or less	2.0~3.7	3.8~4.7	4.8~7.3	7.4 or higher
			6	4.0 or less	4.1~5.1	5.2~6.4	6.5~8.0	8.1 or higher
		Female	5	1.7 or less	1.8~3.1	3.2~4.5	4.6~5.3	5.4 or higher
			6	3.7 or less	3.8~5.1	5.2~5.7	5.8~7.5	7.6 or higher
	Jump (cm)	Male	5	79 or less	80~99	100~109	110~129	130 or higher
			6	92 or less	93~109	110~119	120~138	139 or higher
		Female	5	66 or less	67~84	85~94	95~113	114 or higher
			6	79 or less	80~94	95~107	108~124	125 or higher
	Static balance (sec)	Male	5	2.1 or less	2.2~4.1	4.2~8.5	8.6~23.0	23.1 or higher
			6	2.9 or less	3.0~6.8	6.9~12.3	12.4~26.3	26.4 or higher
		Female	5	2.6 or less	2.7~5.8	5.9~10.1	10.2~24.2	24.3 or higher
			6	2.6 or less	2.7~6.4	6.5~15.1	15.2~49.3	49.4 or higher
	Dynamic balance (sec)	Male	5	31.0 or higher	18.1~30.9	11.0~18.07	7.3~10.9	24.3 or less
			6	25.0 or higher	15.7~24.9	9.3~15.6	6.6~9.2	6.5 or less
		Female	5	30.4 or higher	17.4~30.3	12.7~17.3	9.0~12.6	8.9 or less
			6	30.1 or higher	14.8~30.0	9.9~14.7	5.8~9.8	5.7 or less
	Throw (score)	Male	5	0	1	2~3	4~6	7 or higher
			6	0	1~3	4~5	6~7	8 or higher
		Female	5	0	1	2~3	4~5	6 or higher
			6	0	1~3	4~5	6	7 or higher
	Kick (score)	Male	5	0	1~3	4~6	7~9	10 or higher
			6	2 or less	3~5	6~7	8~9	10 or higher
		Female	5	0	1~3	4~6	7~9	10 or higher
			6	1 or less	2~4	5~6	7~9	10 or higher
Physical fitness habits	Steps (step)	Male	5	3247 or less	3248~5086	5087~6759	6760~9036	9037 or higher
			6					
		Female	5					
			6					
	Exercise time (min)	Male	5	3 or less	4~27	28~39	40~80	90 or higher
			6					
		Female	5					
			6					
	Screen time (min)	Male	5	88 or higher	46~87	34~45	10~33	9 or less
			6					
		Female	5					
			6					
	Sleep time (min)	Male	5	479 or less	480~599	600	780	8401 or higher
			6					
		Female	5					
			6					
Physical fitness attitude	Preference (score)	Male	5	7 or less	8~13	14~15	16~19	20 or higher
			6					
		Female	5					
			6					
	Enjoyment (score)	Male	5	10 or less	11~13	14~16	17~19	20 or higher
			6					
		Female	5					
			6					
	Confidence (score)	Male	5	7 or less	8~12	13~15	16~19	20 or higher
			6					
		Female	5					
			6					

**Table A2:** Physical activity competence evaluation standards (BMI).

Category	Contents	Gender	Years	Phase 3 (Underweight)	Phase 1 (Normal Weight)	Phase 3 (Over Weight)	Phase 4 (Obesity)
Physical fitness for health	Preference (score)	Male	5	13.9 or less	14.0~17.2	17.3~18.5	18.6 or higher
			6	14.0 or less	14.1~17.8	17.9~19.5	19.6 or higher
		Female	5	13.3 or less	13.4~16.8	16.9~18.2	18.3 or higher
			6	13.8 or less	13.9~17.3	17.3~19.4	19.5 or higher
